# The Conference on Tuberculosis

**Published:** 1904-10-01

**Authors:** 


### The Conference on Tuberculosis.

We have received the September number of the "Monthly Publication" of the Central International Bureau for the Prevention of Consumption, with a Supplement containing the Papers presented to the International Tuberculosis Conference held at Copenhagen in May. One of the most important of these deals with the question of compulsory notification of consumption, and is contributed by Dr. Holmboe, of Christiania, who sets forth the results of such notification in Norway, where it has been enforced since May 8th, 1900. In accordance with the law of that date, when a physician for the first time attends a case of tuberculosis "where isolation is necessary," he is obliged to give notice of the case to the president of the (local) Health Commission, who in Norway is always a physician. He has also to report to the same official when a death caused by such a disease occurs in his practice. Furthermore, a report to the Health Commission can be ordered by the municipal authorities, with the consent of the Government, when a patient changes his address. The statistics of the new law for the first year are now complete, and they show notification of 10,944 cases in 1901, of which 10,086 were of lung tuberculosis. It is said that it has been "quite exceptional" to find in the reports of the district physicians any mention of unpleasantness caused to the patients by the notifications, and Dr. Holmboe declares, as the result of Norwegian experience, (1) that the introduction of compulsory notification is desirable in order to combat tuberculosis as an infectious disease, and should be introduced wherever conditions allow. (2) That it should embrace all cases in which isolation is necessary, all cases of death caused by tuberculosis, and all changes of dwelling when such declaration would not be attended with too great difficulties. (3) That experience has shown that compulsory notification, if judiciously carried out with the aid of the sanitary authorities, need by no means come into conflict with humane principles. A full international report upon the same question, by a committee presided over by Dr. Putzeys of Liege, is too long for quotation, but Dr. Holmboe was a member of this committee, and its report is in essential agreement with his conclusions, while, like him, it fails to define with sufficient clearness the class of cases in which "isolation is necessary." It is obvious that early cases of phthisis may be as dangerous to relatives as advanced ones. The "Publication" contains much interesting matter, but it is surely needless, in the present state of education, that every paper should be printed in three languages. The cost of this must, we think, be greatly in excess of the benefit likely to be derived.

